# GPBAR1/TGR5 Mediates Bile Acid-Induced Cytokine Expression in Murine Kupffer Cells

**DOI:** 10.1371/journal.pone.0093567

**Published:** 2014-04-22

**Authors:** Guiyu Lou, Xiaoxiao Ma, Xianghui Fu, Zhipeng Meng, Wenyu Zhang, Yan-Dong Wang, Carl Van Ness, Donna Yu, Rongzhen Xu, Wendong Huang

**Affiliations:** 1 Department of Biochemistry and Molecular Biology, Third Military Medical University, Chongqing, China; 2 Division of Molecular Diabetes Research, Department of Diabetes and Metabolic Diseases Research, Beckman Research Institute, City of Hope National Medical Center, Duarte, California, United States of America; 3 Irell & Manella Graduate School of Biological Sciences, Beckman Research Institute, City of Hope National Medical Center, Duarte, California, United States of America; 4 Department of Hematology and Cancer Institute, Second Affiliated Hospital, School of Medicine, Zhejiang University, Hangzhou, China; Heart Research Institute, Australia

## Abstract

GPBAR1/TGR5 is a novel plasma membrane-bound G protein–coupled bile acid (BA) receptor. BAs are known to induce the expression of inflammatory cytokines in the liver with unknown mechanism. Here we show that without other external stimuli, TGR5 activation alone induced the expression of interleukin 1β (IL-1β) and tumor necrosis factor-α (TNF-α) in murine macrophage cell line RAW264.7 or murine Kupffer cells. The TGR5-mediated increase of pro-inflammatory cytokine expression was suppressed by JNK inhibition. Moreover, the induced pro-inflammatory cytokine expression in mouse liver by 1% cholic acid (CA) diet was blunted in JNK^−/−^ mice. TGR5 activation by its ligands enhanced the phosphorylation levels, DNA-binding and trans-activities of c-Jun and ATF2 transcription factors. Finally, the induced pro-inflammatory cytokine expression in Kupffer cells by TGR5 activation correlated with the suppression of Cholesterol 7α-hydroxylase (Cyp7a1) expression in murine hepatocytes. These results suggest that TGR5 mediates the BA-induced pro-inflammatory cytokine production in murine Kupffer cells through JNK-dependent pathway. This novel role of TGR5 may correlate to the suppression of Cyp7a1 expression in hepatocytes and contribute to the delicate BA feedback regulation.

## Introduction

TGR5 is a plasma membrane-bound G protein–coupled bile acid (BA) receptor, which displays varied levels of expression in different tissues [1]–[3]. Hydrophobic BAs, such as lithocholic acid (LCA) and deoxycholic acid (DCA), are potent endogenous ligands of TGR5. Emerging evidence shows that TGR5 regulates glucose homeostasis, increases energy expenditure in brown adipose tissue and contributes to BA homeostasis [4]–[6]. Another well-defined function of TGR5 is its potent anti-inflammatory effect. Expression of TGR5 is detected in macrophages, including Kupffer cells in the liver [7]. THP-1 cells over-expressing TGR5 suppress the cytokine production induced by lipopolysaccharide (LPS) challenge [1]. In vivo study demonstrates that activation of TGR5 decreases LPS-induced inflammation in the liver [8] as well as inflammation in atherosclerotic plaque [9]. However, the physiological roles of TGR5 in pro-inflammatory cytokine expression without other inflammatory stimulation are still unknown.

The dual function of BAs in inflammation has been previously reported. Studies have shown that DCA and chenodeoxycholic acid exert an inhibitory effect on interleukin 1β (IL-1β), interleukin-6 (IL-6) and tumor necrosis factor-α (TNF-α) production by LPS-stimulated macrophages [10]. Another report demonstrates that BAs can induce the synthesis and excretion of pro-inflammatory cytokines such as TNF-α and IL-1β in hepatic macrophages (Kupffer cells) [11]. These pro-inflammatory cytokines then negatively regulate the expression of Cholesterol 7α-hydroxylase (Cyp7a1), a liver-specific enzyme that catalyzes the first and rate-limiting step in the BA synthetic pathway [11]. These findings raise the question of whether the induction of cytokines in macrophages by BAs in the absence of an additional stimulus is mediated by TGR5, since farnesoid X receptor (FXR), the nuclear receptor of BA, is mainly expressed in hepatocytes [12] in the liver.

The present studies demonstrate that TGR5 is the receptor that mediates the BA-induced pro-inflammatory cytokine production in Kupffer cells through JNK-dependent pathway. Both c-Jun and ATF2 are downstream transcription factors after TGR5 activation to activate pro-inflammatory cytokine expression. This novel role of TGR5 may regulate Cyp7a1 expression and contribute to the delicate BA feedback regulation.

## Materials and Methods

### Reagents

BMS-345541, SP600125 and H89 were purchased from Calbiochem (San Diego, CA). Oleanolic acid (OA) was obtained from Sigma-Aldrich (St. Louis, MO). 8CPT-2Me-cAMP (cAMP analog) was from TOCRIS Bioscience (Ellisville, MO). Brefeldin A (BFA) was purchased from Epicenter Biotechnologies (Madison, WI). 23(s)-MeCDCA(>99% purity) (a TGR5-specific ligand) was made at City of Hope. pCRE-luc and pC/EBP-luc were purchased from Stratagene (Santa Clara, CA). Lipofectamine 2000 and Lipofectamine LTX were from Invitrogen (Carlsbad, CA). siRNA-c-Jun and Hiperfect were from Qiagen (Valencia, CA). siRNA-ATF2 was from Santa Cruz Biotechnology, Inc. (Santa Cruz, CA). Fetal calf serum (low endotoxin) was from Gibco (Birmingham, MI). The Dual-Luciferase Reporter Assay System was purchased from Promega (Madison, WI). Antibodies against JNK (56G8) rabbit monoclonal antibody, phospho-JNK (Thr183/Tyr185), phospho-c-Jun (Ser63) II, c-Jun (60A8) rabbit monoclonal antibody, antibodies for phospho-ATF2 and ATF2 were from Cell Signaling (Danvers, MA). Anti-pATF2, anti-pc-Jun for ChIP, and anti-β-actin were from Santa Cruz Biotechnology, Inc. (Santa Cruz, CA).

### Animal maintenance and treatments

C57BL/6 and c-Jun-N-terminal kinase 1 (JNK1) knockout mice were purchased from The Jackson Laboratory (Bar Harbor, ME). TGR5^−/−^ mice were obtained from Dr. Vassileva Galya at Merck [3]. Mice were maintained in a pathogen-free animal facility under a standard 12:12-h light/dark cycle and were fed standard rodent chow and water ad libitum. All procedures followed the NIH guidelines for the care and use of laboratory animals. The animal study was approved by City of Hope Institutional Animal Care and Use Committee (IACUC). For studies involving cholic acid (CA) feeding, mice (n = 5/group) between 8 and 10 weeks old were fed with either 1% CA as previously described [11] or the control diet for 3 days.

### Isolation and cultivation of Kupffer cells, hepatocytes and peritoneal macrophages

Kupffer cells were isolated from 4- to 5-month-old C57BL/6 mice by collagenase perfusion and 2-step Percoll gradient centrifugation. Hepatocytes were obtained from 8-week-old C57BL/6 mice via the collagenase perfusion method as previously described [8]. Resident macrophages were obtained from the peritoneal cavity of 8-week-old C57BL/6 mice with phosphate buffer [13]. All 3 types of cells were maintained in Dulbecco's modified Eagle's medium (DMEM) supplemented with 10% fetal calf serum and 1% penicillin/streptomycin for 48 hours.

### Stable expression of TGR5 in HEK293 cells

HEK293 cells were cultured in DMEM with 10% fetal calf serum and were kept at 37°C and 5% CO_2_. A mouse TGR5 expression plasmid (pIRESneo3-mTGR5) was transfected into HEK293 cells using Lipofectamine 2000 according to the manufacturer's instructions. After 48 hours, cells were placed in medium containing 500 µg/mL G418 to select for resistant cells. Resistant clones were screened by real-time PCR.

### Measurement of TNF-α levels with ELISA

RAW264.7 cells were stimulated with dimethyl sulfoxide (DMSO 0.1%), 23(s)-MeCDCA (10 µM) or OA (10 µM) for 12 hours. Cell culture supernatants were then collected, and the levels of TNF-α in the cell culture medium were quantified by using an ELISA kit (R&D, Minneapolis, MN) according to the manufacture's instructions. Three replicates of each group were performed.

### Mutation of AP-1 binding sites in the mouse TNF-α promoter reporter

Mutations in the AP-1/CRE construct were generated using a QuikChange (Stratagene) kit. An 876 bp fragment containing the 5′ flanking region from -877 to -1 in the mouse TNF-α promoter was constructed using the pTK-luc reporter plasmid (pmTNF(−877–−1)-luc), which served as the template for constructing the mutations (pmTNF-mut-luc). Primers used to introduce the mutations were 5′CCTTGGTGGAGAAAACCATGTAGACATGTGGAGGAAGCGGTAGTG3′ and 5′CACTACCGCTTCCTCCACATGTCTACATGGTTTTCTCCACCAAGG3′ (mutation sites of AP-1/CRE are represented by the underlined nucleotides).

### Real-time polymerase chain reaction

Total RNAs were isolated using TRI reagents (Molecular Research Center, Cincinnati, OH) according to the manufacturer's instructions. 2 µg total RNA (was used to synthesize cDNA using the SuperScript First-Strand Synthesis System (Invitrogen, San Diego, CA), and the levels of mRNA were quantified by real-time polymerase chain reaction (PCR) using an Applied Biosystems 7300 Real-Time PCR System (Applied Biosystems, Forest City, CA). Primers used for gene analysis were shown in Table S1. The quantities of each test gene and the quantity of the internal control M36b4 were determined from the standard curve using the Applied Biosystems software.

### Transfection

Transient transfection of pCRE-luc or pC/EBP-luc was performed in a 96-well plate. Briefly, 250 ng of pCRE-luc or pC/EBP-luc plasmid and 25 ng of pRL-TK were transfected into parental HEK293 or TGR5-overexpressing HEK293 cells using Lipofectamine 2000. For the TNF-α promoter reporter assay, either HEK293 or TGR5-overexpressing HEK293 cells were transfected with 0.8 µg pmTNF (−877–−1)-luc or pmTNF-mut-luc and 80 ng pRL-TK in a 24-well plate. 24 hours after transfection, cells were treated with either DMSO, 23(s)-MeCDCA (10 µM) or OA (10 µM) respectively for 24 hours. Luciferase activity was assessed using the Dual-Luciferase Reporter Assay System (Promega, Madison, WI) according to the manufacturer's instructions. At least four replicates of each transfection were performed. RAW264.7 cells were transfected with plasmids pmTNF (−877–−1) luc and pmTNF-mut-luc using Lipofectamine LTX reagent according to the manufacturer's instructions. To normalize the transfection efficiency, cells were co-transfected with pRL-TK plasmid. Cell stimulation experiments were performed 24 hours after reporter plasmid transfection. siRNA transfection was carried out in a 12-well tissue culture plate according to a protocol from Qiagen. siRNA-control, siRNA-c-Jun and siRNA-ATF were transfected using Hiperfect. Forty-eight hours after transfection, cells were incubated for 24 hours with TGR5 ligands in fresh DMEM. At least three replicates of each transfection were performed.

### Western blotting

Proteins were separated by 10% denaturing polyacrylamide gel electrophoresis and transferred onto nitrocellulose membranes. After blocking in 5% non-fat milk, the membranes were exposed to specific primary antibodies at a concentration of 1∶1000. Membranes were then washed and exposed to peroxidase-conjugated secondary antibodies (Amersham Bioscience, UK). Immunoblots were imaged using a medical film processor (SRX-101A, Konica Minolta Medical & Graphic Inc, NY).

### Chromatin immunoprecipitation (ChIP)

RAW264.7 cells were stimulated by either DMSO, 23(s)-MeCDCA (20 µM) or OA (20 µM) for 1 hour and cross-linked with formaldehyde. Chromatin immunoprecipitation (ChIP) assays were performed according to the manufacturer's protocols (Upstate Biotechnology). Briefly, cell extracts were sonicated and divided. Antibodies (4 µg) against pATF2, pc-Jun and normal mouse IgG were added for immunoprecipitation. The immunoprecipitated chromatin was recovered and purified. TNF-α promoter DNA was quantified by real-time PCR analysis using primers for the proximal TNF-α promoter (see Table S1). Primers (see Table S1) spanning a region located ∼5.1 kb upstream of the TNF-α transcriptional start site were used as a control [14]. At least three replicates of each group were performed.

### Co-culture experiment

Primary hepatocytes were seeded in 6-well plates and cultured for 24 hours. Culture media was replaced with conditional culture medium from RAW264.7 cells for 12 hours, which were incubated with DMSO or 10 µmol/L OA for 12 hours. Then, total RNA was extracted for Real-Time PCR. Three replicates of each experiment were performed.

### Statistical analysis

All data represent at least two independent experiments and are expressed as the means±SEM. The Student's t-test was used to calculate P values, unless stated otherwise. For multiple comparisons between groups, an analysis of variance (ANOVA), followed by Bonferroni's Post-hoc test, was performed. P value less than 0.05 were considered significant.

## Results

### Activation of TGR5 increases cytokine expression

**Figure 1:**
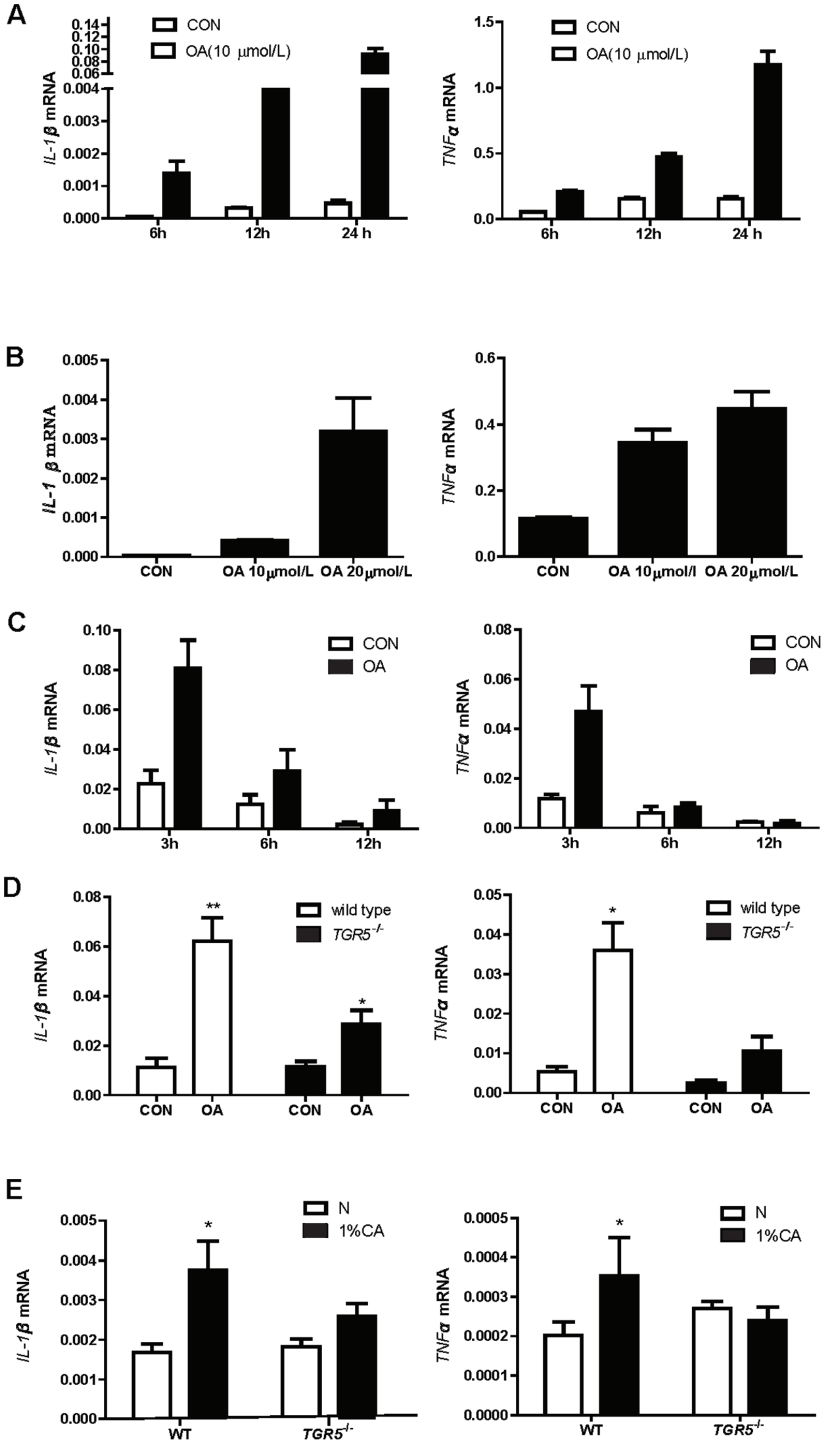
TGR5 activation increases pro-inflammatory cytokine expression in macrophages. A & B. RAW264.7 cells were treated with10 µM OA for different time (A) or at different dose for 24 h (B). C& D Kupffer cells were treated with OA for indicated time or for 3 h. E. Wild-type and TGR5^−/−^ mice (n = 5/group) were fed with normal diet [1] or 1% CA diet for 3 days. Total RNAs from cells and mouse livers were extracted and subjected to RT-PCR analysis. Three independent experiments were performed. Expression of cytokine transcripts was normalized to m36b. Data are expressed as the mean±SEM, ^*^P<0.05 or^**^P<0.01 compared to the controls as determined by one-way ANOVA. P values of panel B were calculated by two-way ANOVA.

We first determined whether expression of cytokine genes was increased when TGR5 was activated. TGR5 is expressed in murine macrophage cell line RAW264.7 (data not shown). Treatment of RAW264.7 cells with TGR5 agonist: oleanolic acid (OA, 10 µM) resulted in strong increase of IL-1β and TNF-α mRNA levels compared to the vehicle control group at 6, 12, and 24 h (Fig.1A). Moreover, these OA effects (24 h) were in a dose-dependent manner (Fig.1B). Similarly, up-regulation of IL-1β and TNF-α expression was also observed in isolated liver Kupffer cells at 3 h by OA (10 µM) treatments (Fig.1C). These results demonstrated that TGR5 activation indeed induced IL-1β and TNF-α expression in both macrophage cell line and Kupffer cells. We also compared the expression levels of these two cytokine induced by OA (10 µM) to LPS (1 ng/mL). The induction of IL-1β by OA was approximate to one fifth of LPS induced expression and almost equal to LPS in the TNF-α expression level (Fig. S1). To determine the specificity of TGR5 in up-regulating cytokine expression, we compared the cytokine expression in wild-type and TGR5^−/−^ Kupffer cells. OA induced much higher IL-1β and TNF-α expression in Kupffer cells from wild-type livers than those from TGR5^−/−^ livers (Fig.1D). The slight effect of OA in TGR5^−/−^ cells could be due to the weak activity of OA on farnesoid X receptor [15] or other proteins. These results thus support a novel role of TGR5 in up-regulating pro-inflammatory cytokine expression in Kupffer cells. To determine the role of TGR5 in mediating BA-induced pro-inflammatory cytokine expression in vivo, we fed the wild-type or TGR5^−/−^ mice with 1% CA diet for 3 days then measured the mRNA levels of IL-1β and TNF-α in the liver. The results showed that mRNA expression of these two cytokines in the liver was significantly increased after 1% CA diet feeding in wild-type but not in TGR5^−/−^ mice (Fig.1E). We did not observe the toxicity of CA to the mouse liver after three days feeding (data not shown). These results thus indicate that TGR5 mediates the BA-induced pro-inflammatory cytokine expression in the liver.

### JNK is involved in mediating the induction of cytokines after TGR5 activation

**Figure 2:**
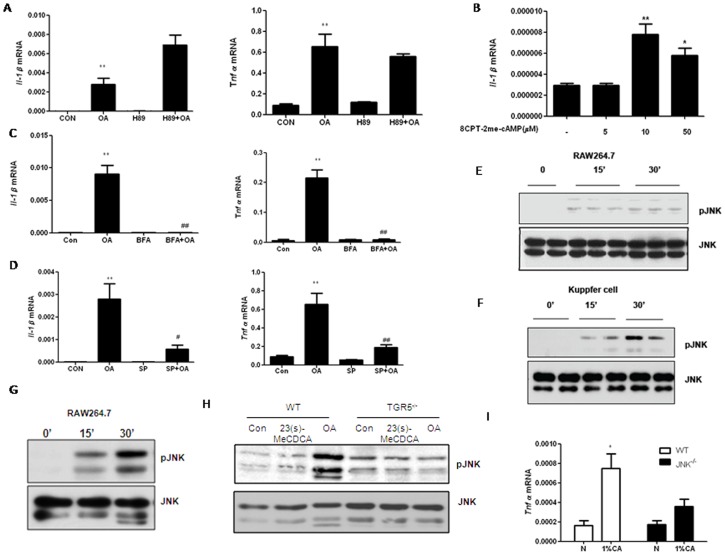
JNK mediates cytokine expression after TGR5 activation. A. RAW264.7 cells were pretreated with 10 µM H89 for 30 min prior to treatment with 10 µM OA for 24 h. B. RAW264.7 cells were treated with different dose of 8CPT-2Me-cAMP for 24 h. 10 µM BFA (C) or SP (D) was incubated with RAW264.7 cells for 30 min before the treatment of 10 µM OA for 24 h. RNA from each treatment was extracted and subjected to RT-PCR analysis. E&F. RAW264.7 cells (E) or Kupffer cells (F) were incubated with 10 µM OA for the indicated time points. G. RAW264.7 cells were incubated with 20 µM 23(s)-MeCDCA for the indicated time points. H. Peritoneal macrophages isolated from mice were incubated with 10 µM OA or 23(s)-MeCDCA for 30 min. The cell lysate was subjected to western blotting analysis for phosphorylation of JNK. Representative western blots are shown. I. Wild-type and JNK^−/−^ mice were fed with normal diet or 1% CA diet for 3 days. Total RNAs from mouse livers (n = 5) were extracted and subjected to RT-PCR analysis. Three independent experiments were performed. Data are expressed as the mean±SEM.^*^P<0.05 or ^**^P<0.01 compared to the controls; ^#^P<0.05 or ^##^P<0.01 compared to the OA as determined by two-way ANOVA. P values of panel B were calculated by one-way ANOVA.

Transducing signal through Gs-protein results in cAMP generation and the subsequent activation of protein kinase A (PKA). Therefore, we asked if PKA is required for TGR5-mediated pro-inflammatory cytokine production. OA-induced expression of IL-1β and TNF-α mRNAs were not inhibited in RAW264.7 cells pretreated with a PKA inhibitor H89 (Fig.2A), suggesting that PKA was not required for cytokine induction by TGR5 activation. H89 inhibited the activity of CREB, the downstream transcription factor of PKA, which indicated that H89 was effective (Fig. S2). Exchange protein directly activated by cAMP (EPAC) was identified as a guanine nucleotide exchange factor for Rap1 that is activated by cAMP in a PKA independent manner [16]. The newly described cAMP analog, 8CPT-2Me-cAMP activates EPAC but not PKA [17]. Treatment of cells with 10 µM CPT-2Me-cAMP led to significant increase IL-1β mRNA expression (Fig.2B). Conversely, if RAW264.7 cells were incubated with an EPAC inhibitor, Brefeldin A, before treatment with OA, the induction of IL-1β and TNF-α mRNA expression was inhibited (Fig.2C). These results indicate that EPAC, but not PKA, may participate in TGR5-mediated pro-inflammatory cytokine induction.

JNK is a member of mitogen activated protein kinases (MAPKs), which can be activated by EPAC [18]. We therefore examined whether inhibition of the JNK pathway could block the TGR5-dependent pro-inflammatory cytokine induction. JNK inhibitor, SP600125 (10 µM), dramatically reduced the OA-induced increase of IL-1β and TNF-α transcription in RAW 264.7 cells (Fig.2D). JNK phosphorylation was increased by OA treatment rapidly in both RAW 264.7 cells (Fig.2E) and Kupffer cells (Fig.2F). The same results were observed by TGR5-specific ligand, 23(s)-MeCDCA in RAW 264.7 cells (Fig.2G) [8]. The increased phosphorylation levels of JNK in wild-type peritoneal macrophages were not observed in TGR5^−/−^ peritoneal macrophages (Fig.2H), which indicated that phosphorylation of JNK was dependent on TGR5 activation. Moreover, TNF-α expression was increased in normal livers but impaired in JNK1^−/−^ livers after CA diet feeding (Fig. 2I). These results demonstrated that JNK was a critical downstream effector in TGR5-mediated cytokine induction in the liver.

Transcription factor nuclear factor-κB (NF-κB) is an important regulator of inflammation and has been shown to inhibit proinflammatory cytokine production by TGR5 activation [9]. Thus, we also examined whether NF-κB stimulation was required for TGR5-mediated pro-inflammatory cytokine induction. Pre-treatment of RAW 264.7 cells with NF-κB inhibitor: BMS-345541 prior to TGR5 activation did not block the OA-induced production of IL-1β and TNF-α mRNAs (Fig. S3). The effect of BMS-345541 on LPS-induced production of IL-1β and TNF-α mRNAs was observed in order to verify the efficiency of BMS-345541 in RAW264.7 cells (Fig. S4). Therefore, the TGR5-mediated increase in cytokine production was NF-κB-independent.

### Activation of TGR5 increases phosphorylation of c-Jun and ATF2

**Figure 3:**
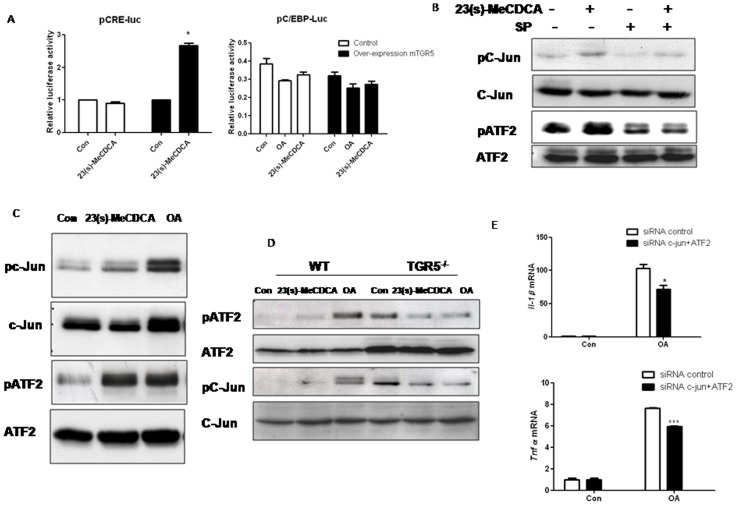
TGR5 activation increases the activities of ATF2 and c-Jun. A. HEK293 cells transfected with pCRE-luc or pC/EBP-luc were treated with 23(s)-MeCDCA (10 µM) for 6 h. Luciferase and Renilla activities were measured and compared. *P<0.05 compared to the controls. B. RAW246.7 cells pretreated with 10 µM SP were treated with 23(s)-MeCDCA (10 µM) for 45 min. The cell lysate was subjected to western blotting analysis. C&D. Kupffer cells (C) or peritoneal macrophages (D) were incubated with 10 µM 23(s)-MeCDCA, OA for 1 h. The cell lysate was subjected to western blotting analysis. E. RAW246.7 cells were transfected with siRNAs then treated with OA. Total RNAs were subjected to RT-PCR analysis. Three independent experiments were performed. Data are expressed as the mean±SEM. ^*^P<0.05, ^***^P<0.001 compared to the controls as determined by two-way ANOVA.

JNK has been shown to activate both ATF2 and c-Jun. To determine whether ATF2 and c-Jun are activated following TGR5 activation, we tested the effects of 23(s)-MeCDCA on the activation of a pCRE-luc reporter vector, which contains the binding sites for ATF2, c-Jun and CREB. 23(s)-MeCDCA treatment led to a 3-fold increase (p<0.05) in CRE-dependent luciferase activity in TGR5-transfected HEK293 cells (Fig.3A). In contrast, there was no significant difference in C/EBP activity, which is not a downstream transcriptional factor of JNK, but also can bind to cytokine promoters (Fig.3A). 23(s)-MeCDCA treatment increased phosphorylation of c-Jun and ATF2, which was blunted by JNK inhibitor, SP600125 (Fig.3B) in RAW264.7 cells. Increased phosphorylation of ATF2 and c-Jun after 23(s)-MeCDCA or OA treatment was only observed in wild-type but not in TGR5^−/−^ peritoneal macrophages (Fig. 3C&D). Moreover, knockdown of c-Jun and ATF2 by siRNA (The efficiency of knockdown was shown in Fig. S5) inhibited OA-induced IL-1β and TNF-α mRNA expression in RAW264.7 cells (Fig.3E). These results suggested that c-Jun and ATF2 were the potential downstream transcription factors that were activated by TGR5.

### Activation of TGR5 increases TNF-α promoter activity

**Figure 4:**
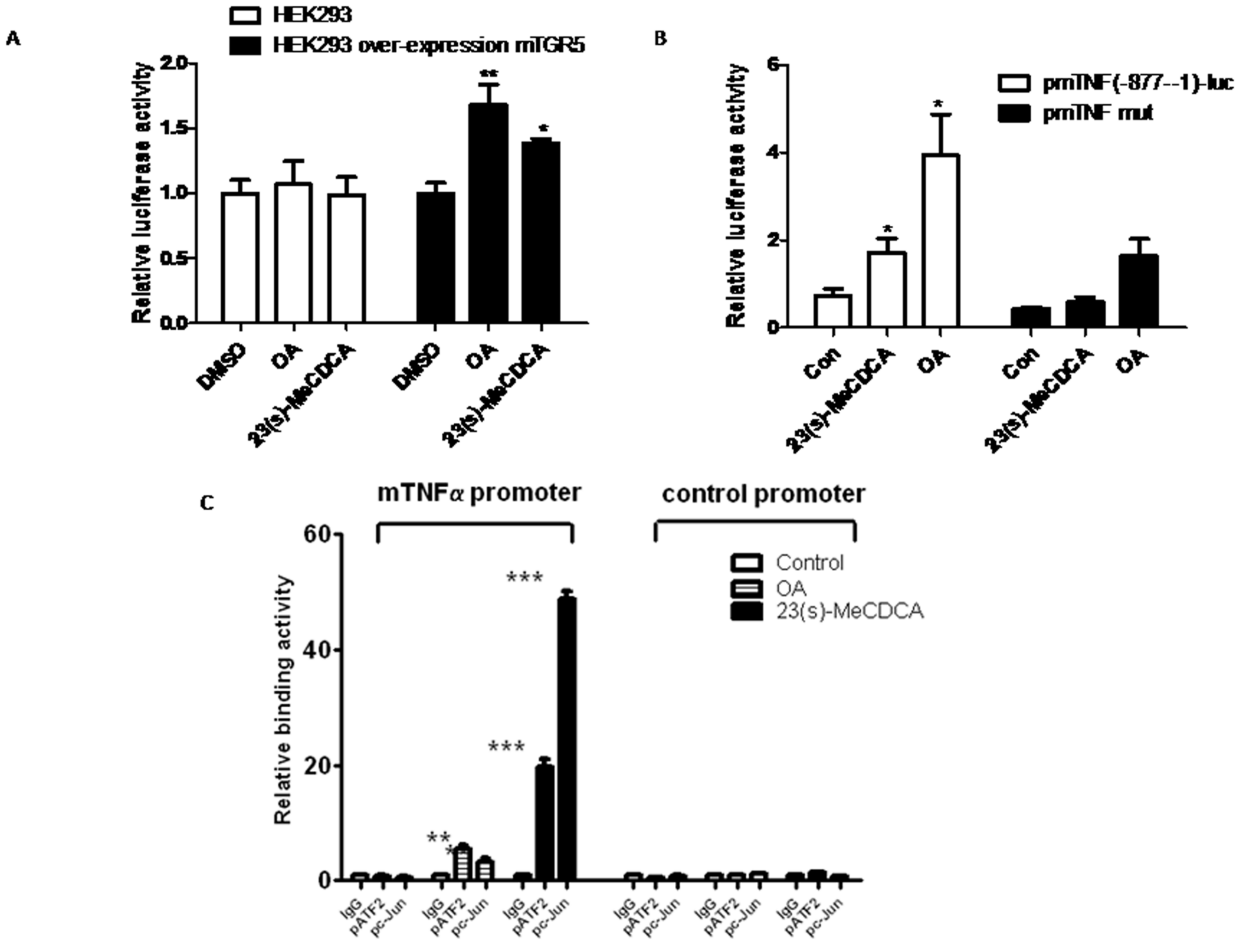
TGR5 activation enhances TNF-α promoter activity. A. pmTNF-α-luc reporter or control plasmid are transfected into cells, then treated with 10 µM 23(s)-MeCDCA or OA for 12 h. Data are expressed as mean±SEM, ^*^P<0.05, ^**^P<0.01 compared to the controls. B. RAW264.7 cells were transfected with pmTNF-α-luc or pmTNF-α-mut-luc reporter constructs, then treated with 23(s)-MeCDCA (10 µM) or OA (10 µM). Cell extracts were prepared and analyzed for luciferase and renilla activities. C. RAW264.7 were treated with 10 µM OA, 20 µM 23(s)-MeCDCA or vehicle control for 1 h. ChIP experiment analysis was performed to measure the binding activity of c-Jun or ATF to the TNF-α promoter. Values are given as fold differences relative to the IgG controls. Two independent experiments were performed. Data are expressed as the mean±SEM. ^*^P<0.05, ^**^P<0.01, ^***^P<0.001 compared to the controls as determined by two-way ANOVA.

Because the promoters of IL-1β and TNF-α contain CRE-binding sites for c-Jun and ATF2 [19], [20], we asked whether promoter activities of TNF-α could be enhanced by OA or 23(s)-MeCDCA treatments. For human TNF-α, the proximal promoter region from -116 to 88 contains adjacent ETS and CRE-like sites. A homologous region exists in mouse gene from -118 to 90. To determine cytokine promoter activity stimulated by 23(s)-MeCDCA or OA, a reporter plasmid encompassing 876 bp nucleotides of mouse TNF-α promoter region was transfected into HEK293 cells. As shown in Fig. 4A, after stimulation by OA or 23(s)-MeCDCA, significant promoter activity was observed in HEK293 cells that were stably transfected with TGR5, but not in parental HEK293 cells, suggesting that TGR5 activation induced TNF-α promoter activity. In contrast, transfection of RAW264.7 cells with a mouse TNF-α promoter containing a mutant CRE binding site did not result in any induction of promoter activity by 23(s)-MeCDCA treatment, although OA slightly activated this reporter (Fig.4B).

To further clarify that the increase of TNF-α promoter activity with TGR5 activation is mediated by JNK pathway, we performed ChIP assays to examine the association between ATF2/c-Jun and TNF-α promoter in RAW264.7 cells by ChIP assays. The results showed that 20 µM 23(s)-MeCDCA or 10 µM OA treatment significantly increased the binding of ATF2 and c-Jun to the TNF-α promoter (Fig.4C). However, no enrichment of these two factors was observed in a genomic region upstream of the TNF-α promoter. Together, these results indicated that TGR5 activation enhanced the recruitment of ATF2 and c-Jun to the TNF-α promoter, thereby increasing TNF-α transcription.

### Activation of TGR5 correlates with the repression of hepatic Cyp7a1 expression

**Figure 5:**
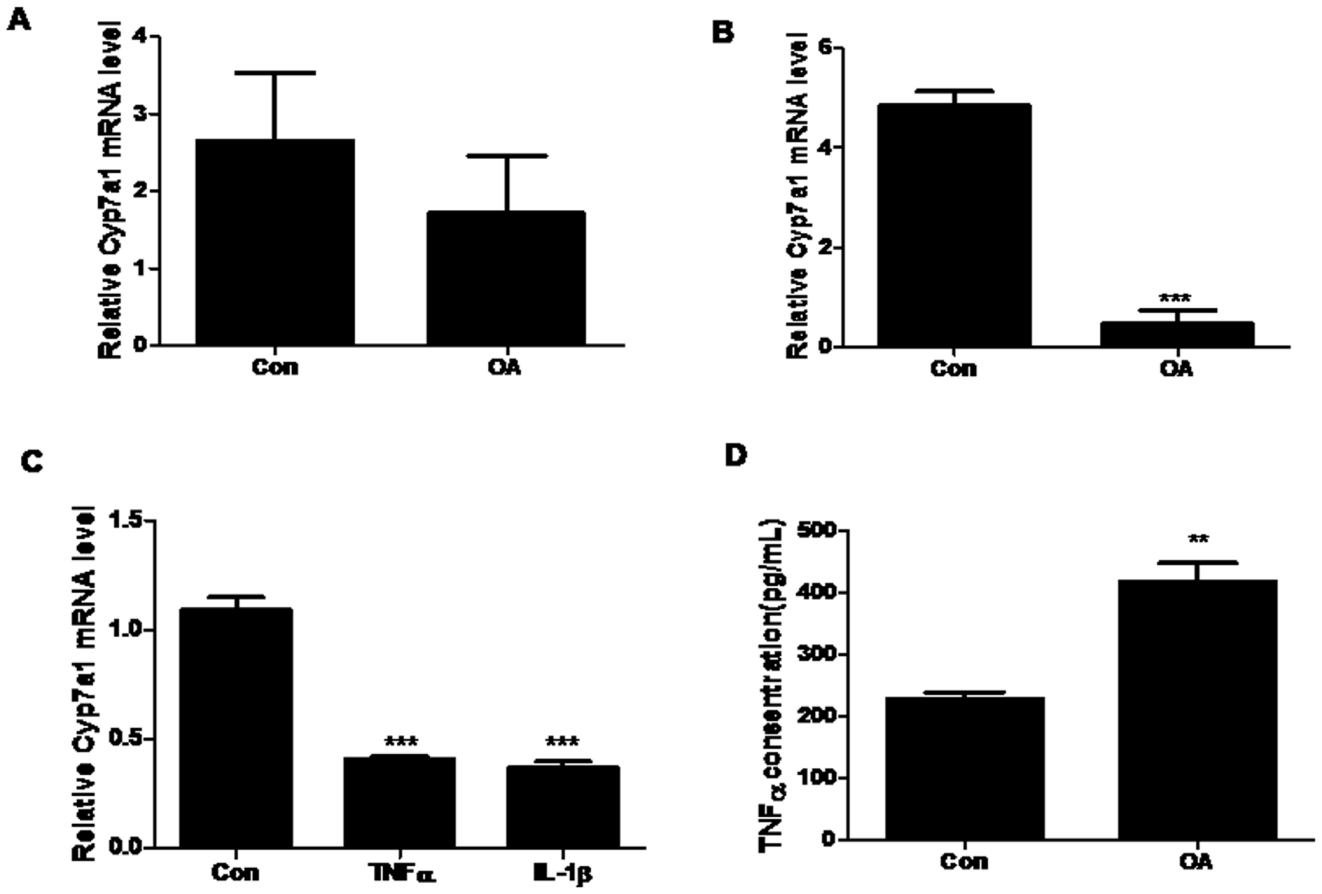
The culture media of RAW264.7 cells treated with OA repress Cyp7a1 expression in hepatocytes. A. Hepatocytes were treated with the IL-1β (1 ng/ml) or TNF-α (10 ng/ml) for 24 h, RNA was prepared and subjected to RT-PCR analysis. M36b4 mRNA was used as a control. ^***^P<0.001 compared to the controls as determined by one-way ANOVA. B&C. Hepatocytes were treated with DMSO or OA for 12 h (B), or the cultured medium of hepatocytes was changed to conditional culture medium from RAW264.7 cells, which were pre-incubated for 12 h with DMSO or OA (C). D. RAW264.7 cells were treated with 10 µmol/L OA for 12 h. TNF-α protein concentration in culture medium was detected by ELISA. Three independent experiments were performed. Data are expressed as the mean±SEM. ^**^P<0.01,^***^P<0.001 compared to the controls as determined by Student's t-test.

Previous studies have shown that cytokines, especially TNF-α and IL-1β, could efficiently suppress Cyp7a1 expression [11]. This was confirmed by our experiments when hepatocytes were incubated with TNF-α or IL-1β (Fig.5A). Therefore, we asked whether TGR5 could also decrease the expression of Cyp7a1 through cytokine induction. Firstly, our results demonstrated that OA treatment had no significant effect on Cyp7a1 expression in the cultured hepatocytes (Fig.5B). We then treated RAW264.7 cells with OA and examined the effects of the conditioned medium on the hepatocyte Cyp7a1 expression. Cyp7a1 mRNA levels in hepatocytes were repressed by 80% when exposed to conditioned medium obtained from RAW264.7 cells after OA treatment (Fig.5C). In order to determine whether the down-regulation of Cyp7a1 by RAW264.7 cells culture medium was due to the increase in cytokine production, TNF-α protein levels in supernatant from RAW264.7 cell culture media were measured. OA treatment at 10 µM produced a significant increase in TNF-α protein levels (p<0.05) compared to the vehicle control (Fig.5D). These results suggested that TGR5 activation induced cytokine expression, which in turn repressed Cyp7a1 expression in hepatocytes.

## Discussion

Activation of TGR5 has been previously shown to inhibit LPS-induced expression of cytokines [7], [9]. In this study, our results demonstrate that activation of the TGR5 by its ligand in the absence of other pro-inflammatory stimuli such as LPS, leads to the up-regulation of IL-1β and TNF-α expression in murine Kupffer cells. This dual role of TGR5 in regulating cytokine expression suggests that TGR5 can modulate inflammatory response through different mechanisms. Examination of the mechanism by which TGR5 activation up-regulates IL-1β and TNF-α expression in murine Kupffer cells indicates that it does not require either PKA or NF-κB, two major regulators of cytokine expression in macrophages. Rather, we show that TGR5-dependent cytokine production is mediated by the activation of JNK. JNK has been reported to mediate the phosphorylation and activation of ATF2 or c-Jun [18], [19]. The ability of ATF2 and c-Jun to up-regulate IL-1β and TNF-α gene expression has also been demonstrated [21]. Therefore, while TGR5 activation inhibits NF-κB-dependent cytokine expression and inflammation induced by LPS, TGR5 activation itself facilitates the activation of JNK pathway to induce cytokine expression in the absence of other inflammatory stimuli. Although increase of intracellular cAMP and PKA activation after TGR5 activation is required in the suppression of LPS-stimulated cytokine production in macrophages. Our results show that activation of EPAC, which is an alternative downstream pathway of cAMP, leads to the production of cytokines by macrophages. Similar to our results, a study shows that TGR5 causes relaxation of gastric smooth muscle by cAMP/EPAC-dependent pathway [22].

Therefore, cAMP suppresses NF-κB-dependent cytokine activation but induces cytokine production through EPAC pathway. This dual effect is also known for other G protein-coupled receptors such as β_2_ adrenergic receptor (β_2_AR) [23]. β_2_AR inhibits NF-κB-dependent cytokine production induced by pro-inflammatory stimuli. However, activation of β_2_AR in the absence of pro-inflammatory stimuli increases IL-1β and IL-6 expression in an ERK1/2- and p38-dependent manner. This may explain that the use of β_2_AR agonists clinically in asthma treatment has both beneficial and detrimental effects, depending on the treatment duration and patient situations. Similarly, our findings indicate that the future use of TGR5 agonists in disease treatments should consider their different effects on cytokine expression.

Previously, Miyake et al have shown that hydrophobic BAs can suppress Cyp7a1 expression via the induction of cytokines in Kupffer cells [11]. Our results suggest that TGR5 could be the endogenous receptor to mediate BA-induced cytokine expression in murine Kupffer cells, which in turn suppresses Cyp7a1 expression in hepatocytes. The levels of BAs are required to be tightly regulated, not only to balance cholesterol levels but also to prevent the potential toxic effects of BAs when they reach abnormally high levels. BAs exhibit negative feedback regulation mainly by repression of Cyp7a1 expression and several mechanisms have been elucidated so far, including FXR/SHP pathway, FXR/fibroblast growth factor15/19 pathway, Pregnane X Receptor (PXR)-mediated pathway, protein kinase C/JNK pathway and cytokine-mediated pathways [24]–[26]. In addition to BAs, cytokines can directly initiate an alternative pathway to suppress Cyp7a1 expression [27]. Moreover, we recently showed that, during acute phase of liver regeneration, Cyp7a1 was also sharply suppressed by hepatocyte growth factor generated after 70% partial hepatectomy [28]. A direct connection between BAs and cytokines may be due to the fact that BAs can activate Kupffer cells to induce cytokine expression to suppress Cyp7a1 transcription. The multiple pathways to control Cyp7a1 expression highlight the importance of BA homeostasis for normal liver functions.

The roles of TGR5 in BA metabolism have been demonstrated in TGR5^−/−^ mice [29]. Our data now suggest that one possible mechanism by which TGR5 controls BA levels maybe through the regulation of Cyp7a1 expression. Therefore, two major BA receptors identified so far may work together to control BA homeostasis. In hepatocytes, FXR may constitute the first and primary level of defense against the toxic effects of BAs by maintaining the normal levels of BAs [30]. In Kupffer cell, TGR5 may establish a secondary and backup pathway for controlling BA levels under physiological conditions. The coordination between FXR and TGR5 in mediating BA effects is not limited to Cyp7a1 expression regulation. Recent studies indicate that both FXR and TGR5 are also required to stimulate gallbladder filling and suppress hepatic inflammation through down-regulation of NF-κB activity [31].

In summary, the present work demonstrates that TGR5 mediates BA-induced expression in murine Kupffer cells, which in turn represses Cyp7a1 transcription in hepatocytes. This novel role of TGR5 may contribute to the delicate regulation of BA homeostasis.

## Supporting Information

Figure S1 **TGR5 activation increases pro-inflammatory cytokine expression in RAW264.7 cells.** RAW264.7 cells were treated with OA (10 µM) or LPS (1 ng/ml) for 24 h. Total RNAs from cells and were extracted and subjected to RT-PCR. Expression of cytokine transcripts was normalized to m36b. Three independent experiments were performed. Data are expressed as the mean±SEM. ^*^P<0.05, ^***^P<0.001 compared to the controls as determined by one-way ANOVA.
(TIFF)

Figure S2 **H89 inhibits the activity of CREB, the downstream transcription factor of PKA.** RAW264.7 cells were exposed to forskolin (10 µM), H89 (10 µM) or forskolin +H89 for 30 min. The cell lysate was subjected to western blotting analysis.
(TIFF)

Figure S3 **NF-κB inhibitor has no effect on OA-induced cytokine expression.** RAW264.7 cells were pre-treated with 5 µM BMS for 30 min prior to treatment with 10 µM OA for 24 h. RNA from each treatment was extracted and subjected to RT-PCR analysis. Three independent experiments were performed. Data are expressed as the mean±SEM. ^***^P<0.001 compared to the controls as determined by one-way ANOVA.
(TIFF)

Figure S4 **NF-κB inhibitor inhibits LPS-induced cytokine expression.** RAW264.7 cells were pretreated with 5 µM BMS for 30 min prior to treatment with 1 ng/ml LPS for 6 h. Total RNAs were extracted and subjected to RT-PCR analysis. Three independent experiments were performed. Data are expressed as the mean±SEM. ^***^P<0.001 compared to the controls; or ^###^P<0.001 compared to LPS as determined by one-way ANOVA.
(TIFF)

Figure S5 **The inhibitory effect of siRNA for c-Jun and ATF2.** RAW264.7 cells were seeded in a 12-well tissue culture plate. siRNA-control, siRNA-c-Jun and siRNA-ATF were transfected using Hiperfect according to the instructions from Qiagen. The cell lysate was subjected to western blotting analysis after 24 h.
(TIFF)

Table S1 **Primer sequences.**
(TIFF)

## References

[1] Kawamata Y, Fujii R, Hosoya M, Harada M, Yoshida H, et al (2003) A G protein-coupled receptor responsive to bile acids. J Biol Chem 278: 9435–9440. 12524422 10.1074/jbc.M209706200

[2] Keitel V, Reinehr R, Gatsios P, Rupprecht C, Gorg B, et al (2003) The G-protein coupled bile salt receptor TGR5 is expressed in liver sinusoidal endothelial cells. Hepatology 45: 695–704. 10.1002/hep.21458 17326144

[3] Vassileva G, Golovko A, Markowitz L, Abbondanzo SJ, Zeng M, et al (2006) Targeted deletion of Gpbar1 protects mice from cholesterol gallstone formation. Biochem J 398: 423–430. 16724960 10.1042/BJ20060537 PMC1559456

[4] Watanabe M, Houten SM, Mataki C, Christoffolete MA, Kim BW, et al (2006) Bile acids induce energy expenditure by promoting intracellular thyroid hormone activation. Nature 439: 484–489. 16400329 10.1038/nature04330

[5] Thomas C, Gioiello A, Noriega L, Strehle A, Oury J, et al (2009) TGR5-mediated bile acid sensing controls glucose homeostasis. Cell Metab 10: 167–177. 19723493 10.1016/j.cmet.2009.08.001 PMC2739652

[6] Maruyama T, Tanaka K, Suzuki J, Miyoshi H, Harada N, et al (2006) Targeted disruption of G protein-coupled bile acid receptor 1 (Gpbar1/M-Bar) in mice. J Endocrinol 191: 197–205. 17065403 10.1677/joe.1.06546

[7] Keitel V, Donner M, Winandy S, Kubitz R, Haussinger D (2008) Expression and function of the bile acid receptor TGR5 in Kupffer cells. Biochem Biophys Res Commun 372: 78–84. 18468513 10.1016/j.bbrc.2008.04.171

[8] Wang YD, Chen WD, Yu D, Forman BM, Huang W (2011) The G-Protein-coupled bile acid receptor, Gpbar1 (TGR5), negatively regulates hepatic inflammatory response through antagonizing nuclear factor kappa light-chain enhancer of activated B cells (NF-kappaB) in mice. Hepatology 54: 1421–1432. 21735468 10.1002/hep.24525 PMC3184183

[9] Pols TW, Nomura M, Harach T, Lo Sasso G, Oosterveer MH, et al (2011) TGR5 activation inhibits atherosclerosis by reducing macrophage inflammation and lipid loading. Cell Metab 14: 747–57. 22152303 10.1016/j.cmet.2011.11.006 PMC3627293

[10] Calmus Y, Guechot J, Podevin P, Bonnefits MT, Giboudeau J, et al (1992) Differential effects of chenodeoxycholic and ursodeoxycholic acids on interleukin 1, interleukin 6 and tumor necrosis factor–α production by monocytes. Hepatology 16: 719–723. 1505915 10.1002/hep.1840160317

[11] Miyake JH, Wang SL, Davis RA (2000) Bile acid induction of cytokine expression by macrophages correlates with repression of hepatic cholesterol 7alpha-hydroxylase. J Biol Chem 275: 21805–21808. 10823815 10.1074/jbc.C000275200

[12] Wang X, Fu X, Van N C, Meng Z, Ma X, et al (2013) Bile Acid Receptors and Liver Cancer. Curr Pathobiol Rep 1: 29–35. 23420103 10.1007/s40139-012-0003-6 PMC3571718

[13] Davies JQ, Gordon S (2005) Isolation and culture of murine macrophages. Methods Mol Biol 290: 91–103. 15361657 10.1385/1-59259-838-2:091

[14] Altmayr F, Jusek G, Holzmann B (2010) J Biol Chem 285: 3525–3531. 20018859 10.1074/jbc.M109.066787 PMC2823491

[15] Liu W, Wong C (2010) Oleanolic acid is a selective farnesoid X receptor modulator. 24: 369–373. 10.1002/ptr.2948 19653193

[16] Ster J, De Bock F, Guerineau NC, Janossy A, Barrere-Lemaire S, et al (2007) Exchange protein activated by cAMP (Epac) mediates cAMP activation of p38 MAPK and modulation of Ca2+-dependent K+ channels in cerebellar neurons. Proc Natl Acad Sci USA 104: 2519–2524. 17284589 10.1073/pnas.0611031104 PMC1892910

[17] Enserink JM, Christensen AE, de Rooij J, van Triest M, Schwede F, et al (2002) A novel Epac-specific cAMP analogue demonstrates independent regulation of Rap1 and ERK. Nat Cell Biol 4: 901–906. 12402047 10.1038/ncb874

[18] Hochbaum D, Tanos T, Ribeiro-Neto F, Altschuler D, Coso OA (2003) Activation of JNK by Epac is independent of its activity as a Rap guanine nucleotide exchanger. J Biol Chem 278: 33738–33746. 12783872 10.1074/jbc.M305208200

[19] Gupta S, Campbell D, Derijard B, Davis RJ (1995) Transcription factor ATF2 regulation by the JNK signal transduction pathway. Science 267: 389–393. 7824938 10.1126/science.7824938

[20] Seimiya H, Mashima T, Toho M, Tsuruo T (1997) c-Jun NH2-terminal kinase-mediated activation of interleukin-1beta converting enzyme/CED-3-like protease during anticancer drug-induced apoptosis. J Biol Chem 272: 4631–4636. 9020192 10.1074/jbc.272.7.4631

[21] Reimold AM, Kim J, Finberg R, Glimcher LH (2001) Decreased immediate inflammatory gene induction in activating transcription factor-2 mutant mice. Int Immunol 13: 241–248. 11157857 10.1093/intimm/13.2.241

[22] Rajagopal S, Kumar DP, Mahavadi S, Bhattacharya S, Zhou R, et al (2013) Activation of G protein-coupled bile acid receptor, TGR5, induces smooth muscle relaxation via both Epac- and PKA-mediated inhibition of RhoA/Rho kinase pathway. Am J Physiol Gastrointest Liver Physiol 304: G527–35. 23275618 10.1152/ajpgi.00388.2012 PMC3602680

[23] Tan KS, Nackley AG, Satterfield K, Maixner W, Diatchenko L, et al (2007) Beta2 adrenergic receptor activation stimulates pro-inflammatory cytokine production in macrophages via PKA- and NF-kappaB-independent mechanisms. Cell Signal 19: 251–260. 16996249 10.1016/j.cellsig.2006.06.007

[24] Stravitz RT, Rao YP, Vlahcevic ZR, Gurley EC, Jarvis WD, et al (1996) Hepatocellular protein kinase C activation by bile acids: implications for regulation of cholesterol 7 alpha-hydroxylase. Am J Physiol 271: G293–303. 8770045 10.1152/ajpgi.1996.271.2.G293

[25] De Fabiani E, Mitro N, Anzulovich AC, Pinelli A, Galli G, et al (2001) The negative effects of bile acids and tumor necrosis factor-alpha on the transcription of cholesterol 7alpha-hydroxylase gene (CYP7A1) converge to hepatic nuclear factor-4: a novel mechanism of feedback regulation of bile acid synthesis mediated by nuclear receptors. J Biol Chem 276: 30708–30716. 11402042 10.1074/jbc.M103270200

[26] Li T, Chiang JY (2005) Mechanism of rifampicin and pregnane X receptor inhibition of human cholesterol 7 alpha-hydroxylase gene transcription. Am J Physiol Gastrointest Liver Physiol 288: G74–84. 15331348 10.1152/ajpgi.00258.2004

[27] Feingold KR, Spady DK, Pollock AS, Moser AH, Grunfeld C (1996) Endotoxin, TNF, and IL-1 decrease cholesterol 7 alpha-hydroxylase mRNA levels and activity. J Lipid Res 37: 223–228. 9026521

[28] Zhang L, Huang X, Meng Z, Dong B, Shiah S, et al (2009) Significance and mechanism of CYP7a1 gene regulation during the acute phase of liver regeneration. Mol Endocrinol 2009 23: 137–145. 10.1210/me.2008-0198 PMC2725763 19056864

[29] Keitel V, Cupisti K, Ullmer C, Knoefel WT, Kubitz R, et al (2009) The membrane-bound bile acid receptor TGR5 is localized in the epithelium of human gallbladders. Hepatology 50: 861–870. 19582812 10.1002/hep.23032

[30] Sina CJ, Tohkin M, Miyata M, Ward JM, Lambert G, et al (2000) Targeted disruption of the nuclear receptor FXR/BAR impairs bile acid and lipid homeostasis. Cell 102: 731–744. 11030617 10.1016/s0092-8674(00)00062-3

[31] Li T, Holmstrom SR, Kir S, Umetani M, Schmidt DR, et al (2011) The G protein-coupled bile acid receptor, TGR5, stimulates gallbladder filling. Mol Endocrinol 25: 1066–1071. 21454404 10.1210/me.2010-0460 PMC3100601

