# Supplementary figures and images for: GPBAR1/TGR5 Mediates Bile Acid-Induced Cytokine Expression in Murine Kupffer Cells
Source: PLoS One. 2014 Apr 22;9(4):e93567. doi: 10.1371/journal.pone.0093567 (PMC3995640; doi:10.1371/journal.pone.0093567)

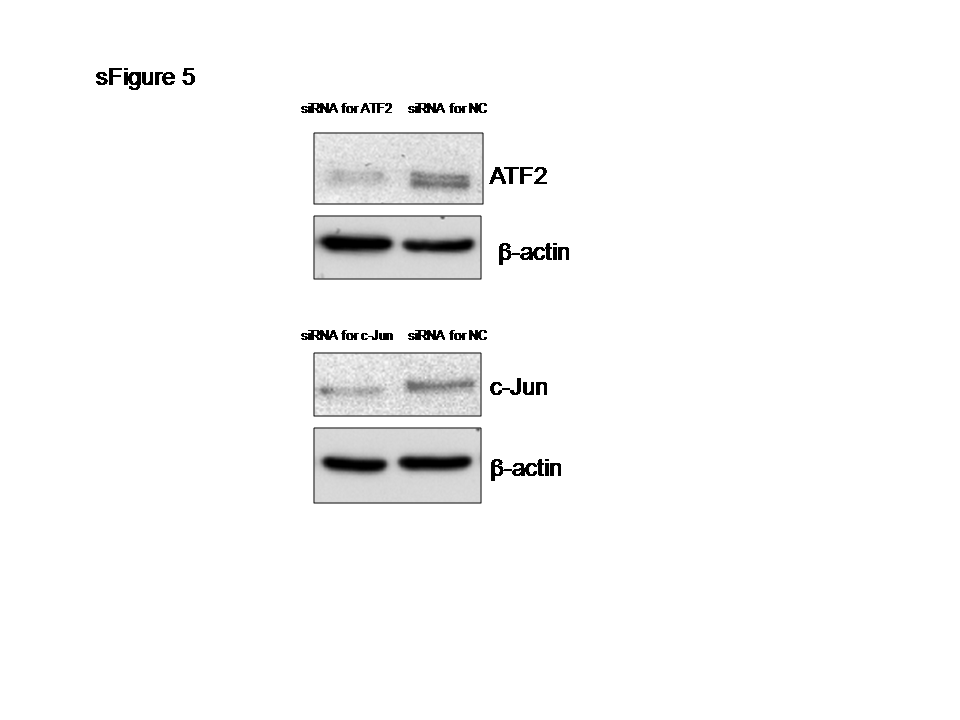

Supplement: Figure S5 — The inhibitory effect of siRNA for c-Jun and ATF2. RAW264.7 cells were seeded in a 12-well tissue culture plate. siRNA-control, siRNA-c-Jun and siRNA-ATF were transfected using Hiperfect according to the instructions from Qiagen. The cell lysate was subjected to western blotting analysis after 24 h. (TIFF) [file pone.0093567.s005.tiff]
